# Severe Natural Outbreak of *Cryptocaryon irritans* in Gilthead Seabream Produces Leukocyte Mobilization and Innate Immunity at the Gill Tissue

**DOI:** 10.3390/ijms23020937

**Published:** 2022-01-15

**Authors:** Laura Cervera, Carmen González-Fernández, Marta Arizcun, Alberto Cuesta, Elena Chaves-Pozo

**Affiliations:** 1 Oceanographic Center of Murcia, Spanish Institute of Oceanography, Spanish National Research Council (IEO-CSIC), Carretera de la Azohía s/n. Puerto de Mazarrón, 30860 Murcia, Spain; laura.cerveram@um.es (L.C.); marta.arizcun@ieo.es (M.A.); 2 Immunobiology for Aquaculture Group, Department of Cell Biology and Histology, Faculty of Biology, Regional Campus of International Excellence “Campus Mare Nostrum”, University of Murcia, 30100 Murcia, Spain; carmen.gonzalez1@um.es (C.G.-F.); alcuesta@um.es (A.C.)

**Keywords:** *Cryptocaryon irritans*, *Sparus aurata*, innate immunity, AMPs, cell-mediated cytotoxicity, aquaculture

## Abstract

The protozoan parasite *Cryptocaryon irritans* causes marine white spot disease in a wide range of fish hosts, including gilthead seabream, a very sensitive species with great economic importance in the Mediterranean area. Thus, we aimed to evaluate the immunity of gilthead seabream after a severe natural outbreak of *C. irritans*. Morphological alterations and immune cell appearance in the gills were studied by light microscopy and immunohistochemical staining. The expression of several immune-related genes in the gills and head kidney were studied by qPCR, including inflammatory and immune cell markers, antimicrobial peptides (AMP), and cell-mediated cytotoxicity (CMC) molecules. Serum humoral innate immune activities were also assayed. Fish mortality reached 100% 8 days after the appearance of the *C. irritans* episode. Gill filaments were engrossed and packed without any space between filaments and included parasites and large numbers of undifferentiated and immune cells, namely acidophilic granulocytes. Our data suggest leukocyte mobilization from the head kidney, while the gills show the up-regulated transcription of inflammatory, AMPs, and CMC-related molecules. Meanwhile, only serum bactericidal activity was increased upon infection. A potent local innate immune response in the gills, probably orchestrated by AMPs and CMC, is triggered by a severe natural outbreak of *C. irritans*.

## 1. Introduction

Nowadays, aquaculture provides nearly 52 percent of the fish for human consumption and represents the most productive food sector [1]. Gilthead seabream (*Sparus aurata* L.) is one of the most produced species in the Mediterranean area and is highly traded with more prosperous markets [2]. However, gilthead seabream cultures still have to overcome certain problems, including diseases. Thus, seabream is very susceptible to *Cryptocaryon irritans*, which causes the death of juveniles within 1 or 2 weeks [3]. *C. irritans* is a holotrich ciliate protozoan that causes marine white spot disease or “marine ich” [4] and is considered to be the most devastating parasitic disease in both mariculture and in ornamental fish, commonly occurring when temperatures are above 19 °C, mainly between 20–30 °C, and generally between June and October [3,5,6,7,8,9,10]. *C. irritans* exhibits very low host specificity and is able to infect multiple fish species, with gilthead seabream being the most affected amongst the cultured species in the Mediterranean area [10]. *C. irritans* invades fish epithelium of the skin, gills, and eyes, altering their physiological functions [3,5,11]. The main signs of cryptocaryoniasis involve the formation of whitish blisters on the skin and eyes, skin discoloration, anorexia, respiratory distress due to excessive mucus secretion, and a disruption of the lamellar structure of the gills, which may appear to be paler [11,12]. During its life cycle, the infective stage, the theront, infects the fish epithelial layer and develops to a trophont stage, which feeds on tissue debris and body fluids, matures, and leaves the host, becoming tomont cells, the external stage [3,9,11].

Economical losses produced by parasites are a matter of concern nowadays. In fact, in China, natural outbreaks of *C. irritans* are quite frequent, causing an estimated USD 100 million in economic losses annually [10]. Natural outbreaks of *C. irritans* have been described in all continents [10], and it is known these outbreaks have a significant impact though their quantification in the aquaculture sector economy worldwide remains unknown. Therefore, knowledge of fish immune response against parasites is crucial to overcome this problem. However, the great diversity and biological differences between parasites, the different immune responses triggered by the hosts [13,14], and the difficulties with their culture in properly performing experimental infections seriously hamper our knowledge about parasite–host interactions. Interestingly, very different types of parasites have evolved ways of evading the immune response [15], increasing the inefficiency of palliative and preventive treatments, and no effective vaccines are available for farmers. Some parasitic infections cause hyperplasia of the target tissue and induce leukocyte mobilization, producing neutrophilia and lymphocytosis, and a local inflammatory response [14]. Macrophages, granulocytes, and mast and rodlet cells are usually involved in the inflammatory response against parasites [13,14]. Inflammation appears to have a key role during *C. irritans* infection, as the transcription of pro-inflammatory cytokines and some molecules of the Toll-like receptor (TLR) signalling pathways, such as interleukin (IL)-1β, IL-8, TLR-2, and myeloid differentiation factor 88 (MyD88), are up-regulated upon infection [7,16]. However, as far as *C. irritans* infections are concerned, there are no studies characterizing the cell types recruited to the site of infection upon a *C. irritants* infection. 

In addition to the inflammatory response, the innate immune response is also orchestrated by antimicrobial peptides (AMP) and cell-mediated cytotoxicity (CMC), among others. Regarding AMPs, few available observations indicate that hepcidin, NK-lysin (NKL), and lysozymes seem to be involved in the immune response against parasites. Thus, the transcription of the gene that codes for hepcidin, named *hamp*, is up-regulated in several fish species upon parasite infection, including *C. irritans* infection [17,18,19,20]. Moreover, NKL, a well-known AMP produced by cytotoxic T-lymphocytes (CTL), has shown antiparasitic effects in turbot (*Scophthalmus maximus*) infected with the ciliate *Philasterides dicentrarchi*, and in yellow croaker (*Larimichthys crocea*) specimens infected with *C. irritans* [21,22]. Lysozymes have also been described to mediate protection against parasites [13]. With respect to the innate CMC, this is a key arm against parasite infections in teleost, and it is played by the natural cytotoxic cells (NCC), which are equivalent to natural killer (NK) cells [23,24]. NCCs express a novel type III membrane protein called NCC receptor protein-1 (NCCRP-1), which binds specifically to an antigen (natural killer target antigen/NKTag) found on protozoan parasites [25,26]. Unfortunately, very little information is available at this regard. The only study regarding the NCC activity in gilthead seabream reports an increase in this activity in specimens infected with the myxozoan parasite *Enteromyxum leei* [27], while the transcription of genes related to the CMC activity such as granzymes and perforin were also up-regulated [24]. Very interestingly, the functional characterization of Nile tilapia (*Oreochromis niloticus*) NCCs demonstrated that they kill ciliate parasites from the genus *Tetrahymena* in a granule-independent mechanism via the Fas ligand (FasL) [25].

Parasites also trigger the adaptive immune response in fish. Thus, some fish species have been immunized against *C. irritants* using experimental vaccines that have conferred partial protection under low levels of exposure [28]. However, there are scarce amount of data about the molecular and cellular characterization of this response and nothing regarding natural infections, where the time of infection and parasite load are unknown and uncontrolled. The main cellular effectors of the adaptive immune response are T and B cells. It is known that a heavy infection with some parasites trigger strong parasite-specific IgM responses [29]. In fact, IgM^+^ B cell proliferation has been described in the head kidney (HK) of rainbow trout (*Oncorhynchus mykiss*) specimens that had been infected with the ciliate *Ichthyophthirius multifiliis* [30]. Moreover, IgT is associated with mucosal immunity [31], and its levels were increased in the gill mucus of survivor specimens from *I. multifiliis* infection [30]. In addition, IgT titres in the intestine are also enhanced when a gut parasite infection occurs [32]. 

Taking the little knowledge available on fish immune response against *C. irritans* and the importance and susceptibility of gilthead seabream into consideration, we carried out the characterization of the immune response upon a natural infection of *C. irritans*. Thus, after the identification of *C. irritans* in the gills as the causative agent of the severe natural outbreak, we evaluated gill histology, leukocyte mobilization, and transcription of immune-related genes in both the gill and HK, in the main hematopoietic tissue, as well as in some serum innate immune responses. 

## 2. Results

### 2.1. Natural Outbreak of Cryptocaryon irritans Produced Gilthead Seabream Mortality

**Figure 1:**
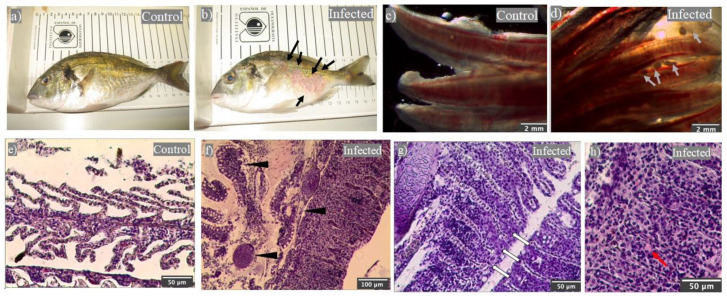
*Cryptocaryon irritans* trophonts are observed in the gills of gilthead seabream under a severe natural infection and produced histopathological alterations. Macroscopical representative images of healthy (Control: (**a**)) and *C. irritans*-infected (**b**) gilthead seabream. Binocular loupe representative images of gills from control (**c**) and *C. irritans*-infected (**d**) gilthead seabream. Histopathological study by light microscopy of gill sections stained with haematoxylin–eosin from control (**e**) and a *C. irritans*-infected (**f**–**h**) gilthead seabream. Note the parasite adhered to the gills (**f**). Scale bar: 2 mm (**c**,**d**) 50 μm (**e**,**g**,**h**), 100 μm (**f**). Black arrows point to skin injuries during *C. irritans* infection. Grey arrows and black arrow heads point to *C. irritans* trophonts. White arrows point to the undifferentiated tissue between the secondary lamellae. Red arrows point to eosinophilic cells.

In July of 2019, we found a sudden mortality episode in our facilities. The mortalities continued over the following 8 days until 100% mortality was reached. Death specimens showed severe skin injuries, mainly ulcers and hemorrhages, which were not present in the control group (Figure 1a,b). The control group was formed by fish from the same batch of hatched eggs (same age) but that were located in other tanks with a controlled temperature at 20 °C.

Since the suspected agent was the parasite *C. irritans*, a conclusion based on historical reports and the water temperature, we evaluated its presence in fresh gill preparations under a microscope. Parasites in the trophont stage were observed in all of the fish specimens from the tank with deaths, whilst it was not observed in any of the control group specimens (Figure 1c,d). Once the presence of *C. irritans* was confirmed, live fish specimens were sampled two days after the onset of death for histological and immunity studies. 

Firstly, a histopathological examination of the gills between the parasitized and control specimens showed important differences. Compared to the controls (Figure 1e), we could observe the parasites embedded in the gill epithelium and that were firmly adhered to the branchial filaments (Figure 1f). The secondary lamellae near the parasites (Figure 1f) showed a great lamellar disorder and engrossed the secondary filaments with undifferentiated cells and leukocytes similar to the cells that formed the deeper layer of the filament epithelium (Figure 1g,h). The capillary spaces normally located in the middle of a very thin epithelium of columnar cells, through which erythrocytes flow, were observed between this dense tissue at regular intervals (Figure 1g,h). Some of these cells were globular in shape, with a small eccentric nucleus and no granules on them but with slight eosinophilic staining (Figure 1h).

### 2.2. Leukocytes, Namely Acidophilic Granulocytes, Are Mobilized upon Infection

**Figure 2:**
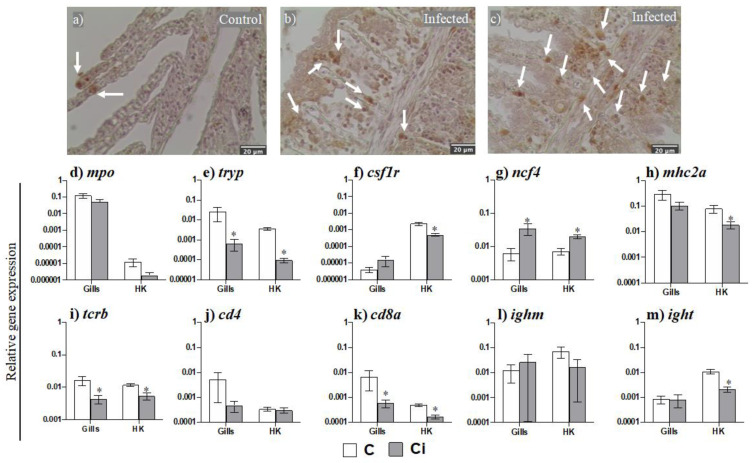
Leukocytes, mainly acidophilic granulocytes, are mobilized upon *Cryptocaryon irritans* infection. Representative gill sections from control (**a**) and *C. irritans*-infected (**b**,**c**) gilthead seabream, immunostained with the D2 mAb. Scale bar = 20 μm (**a**–**c**). White arrows point to AGs. Transcription levels of several leukocyte receptors (**d**–**m**) in the gills and head kidney (HK) from control (C) and *C. irritans*-infected (Ci) gilthead seabream after 2 days of a natural outbreak. Data represent the mean relative gene expression corrected by the *ef1a* expression in each sample ± SEM (*n* = 6) obtained by real-time PCR. (*) Asterisks indicate a significant difference between control and infected specimens according to Student’s *t* test (*p* ≤ 0.05).

We evaluated the potential leukocyte mobilization from the head kidney, the main lympho-hematopoietic tissue in the fish, and the gills upon *C. irritans* parasitation. Firstly, we evaluated the gill presence of acidophilic granulocytes (AGs) by immunohistochemical staining with D2 mAb. AGs were scarcely present in the blood vessels of the secondary filaments in the seabream control specimens (Figure 2a); in the gills from infected specimens, their presence was increased and was largely located in the tissue between the secondary lamella filaments and was even determined to be gathered in clusters (Figure 2b,c). 

We further evaluated leukocyte presence by the transcription of their cellular markers (Figure 2d–m). The transcription of the marker for mast cells (*tryp*) was significantly reduced (Figure 2e) in both the gills and HK upon parasitation, whilst the marker for AGs (*ncf4*) was increased (Figure 2g). Although the marker for the phagocytes (*mpo*) was not altered in any tissue to a significant extent (Figure 2d), that of the macrophages (*csf1r*) (Figure 2f) and antigen-presenting cells (*mhc2a*) (Figure 2h) was only down-regulated in the HK. Regarding the lymphocyte markers, the transcription of the T cells, *tcrb,* and *cd8a* (Figure 2i,k) was down-regulated during infection in both the gills and in the HK, whilst the marker *cd4* was not altered (Figure 2j). In the case of B lymphocytes, *ighm* transcription (Figure 2l) was unaltered whilst that of *ight* (Figure 2m) was only down-regulated in the HK.

### 2.3. C. irritans Infection Produces Inflammation and Activation of the AMP and CMC Responses in the Gills

**Figure 3:**
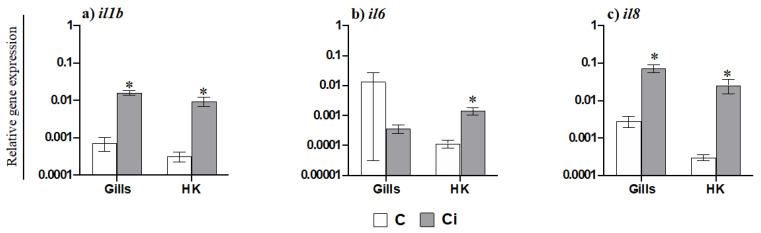
*Cryptocaryon irritans* infection produces inflammation in gilthead seabream tissues. Transcription of inflammatory cytokines (**a**–**c**) in the gills and head kidney (HK) from the control (C) and *C. irritans*-infected (Ci) gilthead seabream after 2 days of a natural outbreak. Data represent the mean relative gene expression corrected by the *ef1a* expression in each sample ± SEM (*n* = 6) obtained by real-time PCR. (*) Asterisks indicate a significant difference between control and infected specimens according to Student’s *t* test (*p* ≤ 0.05).

**Figure 4:**
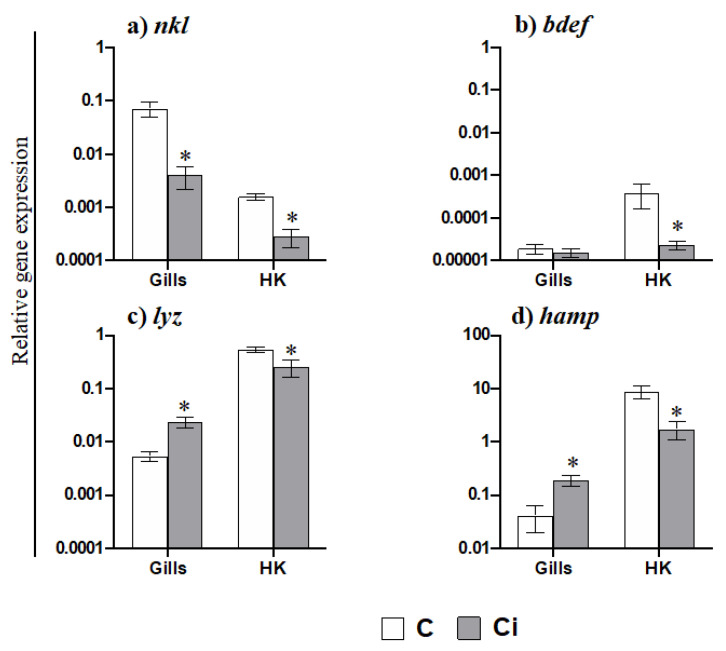
AMPs are down-regulated in the head kidney, but some are increased in the gills upon *Cryptocaryon irritans* infection. Transcription of antimicrobial peptides (**a**–**d**) in the gills and head kidney (HK) from control (C) and *C. irritans*-infected (Ci) gilthead seabream after 2 days of a natural outbreak. Data represent the mean relative gene expression corrected by the *ef1a* expression in each sample ± SEM (*n* = 6) obtained by real-time PCR. (*) Asterisks indicate a significant difference between control and infected specimens according to Student’s *t* test (*p* ≤ 0.05).

**Figure 5:**
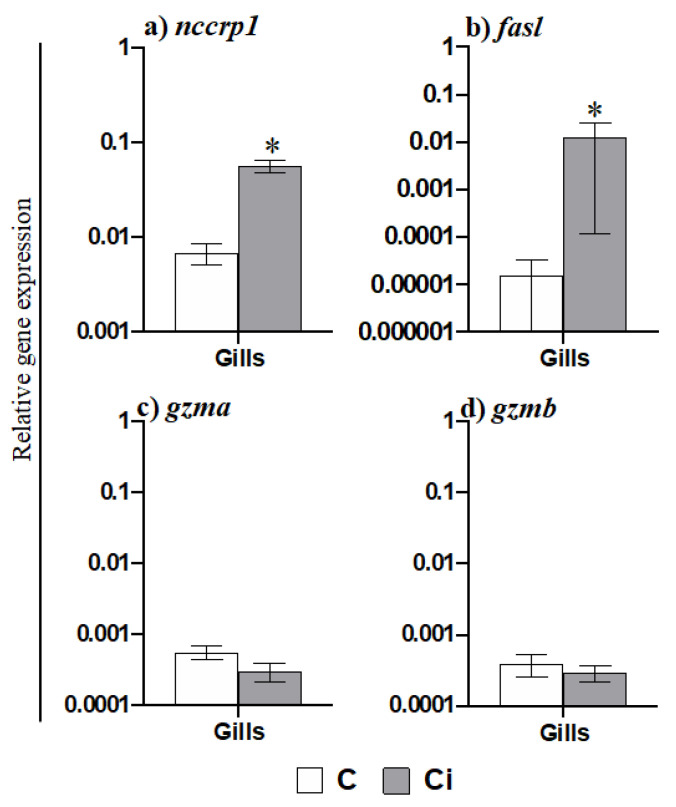
Natural cytotoxic cells, but not granzymes, are involved in the response against *Cryptocaryon irritans*. Transcription of genes related to the cell-mediated cytotoxicity (**a**–**d**) in the gills from control (C) or *C. irritans*-infected (Ci) gilthead seabream after 2 days of a natural outbreak. Data represent the mean relative gene expression corrected by the *ef1a* expression in each sample ± SEM (*n* = 6) obtained by real-time PCR. (*) Asterisks indicate a significant difference between control and infected specimens according to Student’s *t* test (*p* ≤ 0.05).

AGs are the most abundant and important phagocytic and antigen presenting cells in gilthead seabream and are functionally equivalent to mammalian neutrophils. Taking this into account and based on the data obtained regarding the mobilization of AGs to the gills, we further evaluated the transcription of pro-inflammatory cytokines (Figure 3). Thus, the gene expression of *il1b* (Figure 3a) and of the chemokine *il8* (Figure 3c) was highly increased in the gills of fish infected with *C. irritans*, but of the gene expression of *il6* was not increased (Figure 3b). In the HK, however, the transcription levels of *il1b*, *il6,* and *il8* were up-regulated upon infection (Figure 3a–c). In addition, the transcription of several AMP genes was altered in the gills and HK of infected fish compared to in the controls (Figure 4). Thus, while *nkl* expression was down-regulated in the gills of parasitized seabream specimens (Figure 4a), the *lyz* and *hamp* genes were up-regulated (Figure 4c,d). By contrast, all of the AMP genes were down-regulated in the HK (Figure 4a–d). Finally, we also evaluated the transcription of the genes related to the CMC response (Figure 5). Interestingly, we observed that *nccrp1* and *fasl* gene expression was greatly up-regulated in the gills of the infected fish (Figure 5a,b), although other related molecules such as the granzymes *gzma* and *gzmb* were unaltered upon infection (Figure 5c,d). 

### 2.4. Infection with C. irritans Produces Little Changes in the Serum Innate Immunity but Increases the Bactericidal Activity 

**Figure 6:**
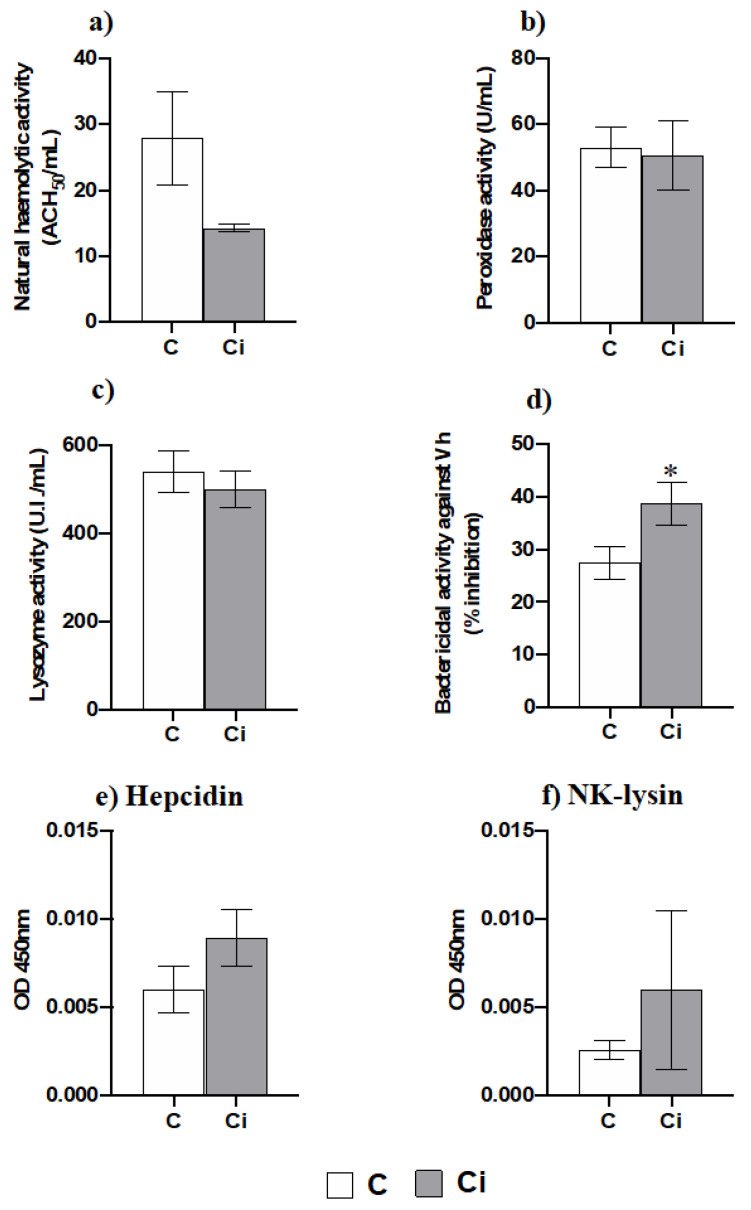
Serum bactericidal activity is increased by infection with *Cryptocaryon irritans***.** Natural haemolytic complement activity (**a**), peroxidase activity (**b**), lysozyme activity (**c**), bactericidal activity against Vh (**d**) as well as Hepcidin (**e**) and NK-lysin (**f**) levels in the serum from control (C) or *C. irritans*-infected (Ci) gilthead seabream after 2 days of a natural outbreak. Data represent the ±SEM (*n* = 6). (*) Asterisks indicate a significant difference between control and infected specimens according to Student’s *t* test (*p* ≤ 0.05).

The serum protein levels were significantly lower in the infected group than they were in the control one according to Student’s t test (Control: 51.358 ± 7.951 mg/mL; Infected: 23.825 ± 3.568 mg/mL; *p* < 0.05). Interestingly, amongst the antimicrobial functions analysed upon *C. irritans* infection (Figure 6a–d), only the serum bactericidal activity was increased (Figure 6d), while the natural haemolytic complement, lysozyme, and peroxidase activities were not affected by the infection (Figure 6a–c). The serum levels of hepcidin and NK-lysin, two important antimicrobial peptides, were unmodified upon infection (Figure 6e,f).

## 3. Discussion

*C. irritans* is a marine parasite that affects gilthead seabream [3], among other species, with asymptomatic to severe stages and can produce massive deaths depending on the environmental and culture conditions. Ciliates in general, and *C. irritans* in particular, are known to affect entire confined populations in few days and have demonstrated high mortality rates [5,11,12], as we have observed in the present study. Thus, once mortalities appeared, no survivors remained 8 days later. We used a group of fish from the same batch of hatched eggs (same age) located in other tank with a controlled temperature at 20 °C as a control group to guarantee the non-infection status, as *C irritans* infects when temperatures are over 20 °C [3,5,10]. Although the difference in temperature between the infected and control fish might alter the data, we decided to use this control since other possible controls for a natural outbreak such as the use of fish of different sizes or those who were asymptomatic from the same tank could also alter the data and would present limitations. In the first case, the different ages might have had an important impact. In the second case, the control fish are part of resistant or survivor groups, probably due to enhanced immunity. Moreover, in recent studies dealing with seasonal changes in the metabolism and cellular stress of gilthead seabream throughout the year, most of the parameters showed differences when compared at the lowest temperatures (February; around 14 °C) but not between fish at 18 or 26 °C [33]. Interestingly, in a laboratory experiment in which the rearing temperature of gilthead seabream was changed for 10 days, the only temperature that showed a physiological alteration compared to fish reared at 18 °C was 30 °C but not at 22, 24, or 26 °C [34]. In this regard, another study [35] evaluated the differential susceptibility of gilthead seabream to *Enteromyxum leei* infection at different temperatures. They found that at 25.6 °C, all of the fish were infected, while at 18 °C, only 58.3% were infected. Interestingly, antibody levels were also the highest at 25.6 °C, though this could be due to either the higher temperature or to the higher parasite load. Taking all of this into account, we could hypothesize that most of the changes observed in this manuscript are due to the presence of the *C. irritants* parasites in the infected fish, though we cannot discard the certain role of the temperature.

One of the most relevant external clinical signs is the presence of skin lesions and ulcers in infected specimens [3], as we have also showed in this study, though these could be due to other factors. Skin lesions might be a consequence of the inflammatory processes triggered by the parasite at the moment of invasion [8,36], and skin ulcerations could appear when the trophont tries to exit the host epithelia, leading to epithelia erosion [17]. Gills are another target site for *C. irritans* [3]. In this work, we have proven that *C. irritans* trophonts are abundant and were firmly adhered to gill epithelium in the infected group, as previously described [11]. The gills are often pale with lamellae that are clumped together. The structural disruption of the gill lamellae during infection has been suggested to cause mucus hyperproduction [11]. However, what we observed in parasitized gilthead seabream is a hypertrophy of the deep layer of the filament epithelium, resulting in gill tissue where the spaces between the secondary lamellae are almost absent. The deep layer of the filament epithelium is formed by a net of undifferentiated neuroendocrine and mainly immune cells, including monocyte-macrophages and granulocytes [37]. Unfortunately, little is known about gill immunity upon *C. irritans* parasitation. 

Due to the observed gill engrossment and the abundance of leukocytes, we have analysed the expression levels of the marker genes for mast cells, macrophages, and acidophilic granulocytes (equivalent in function to neutrophils in seabream). We found that the gene marker for mast cells (*tryp*) was down-regulated while the gene markers for acidophilic granulocytes (*ncf4*) or macrophages (*csf1r*) were up-regulated in the gills of parasitized seabream, though *csf1r* did not reach significance. Regarding mast cells, the authors pointed to an increase in the number of mast cells, which are crucial to fight against parasites [38]; however, other studies failed to find differences in the mast cells in the gills of seabream infected with the copepod *Ergasilus* sp., in European sea bass (*Dicentrarchus labrax*) infected with the monogenean *Diplectanum aequans,* or in the gut of European perch (*Perca fluviatilis*) infected with an enteric worm [38,39,40,41]. Regarding the AGs, the increase in this cell type in the gills suggested by the *ncf4* transcription was further confirmed using a specific antibody (D2) [42]. In gilthead seabream, the acidophilic granulocytes are quickly mobilised upon infection (24 h after bacterial infection) [43]. In fact, AG infiltration has been related to an increase in *il8* gene expression [44]. Severe inflammatory response after *C. irritans* infection has been previously described in different species of fish [17,45,46,47]. In our study, as in other studies, the *il1b* expression levels were up-regulated in the gills and HK upon *C. irritans* infection [7,48]. In addition, *il1b* can act as an inducer of other cytokines with pro-inflammatory and chemotactic functions, such as *il8* [13]. In our study *il8* gene expression was strongly up-regulated in both the gills and in the HK during *C. irritans* infection, something that also occurs in grouper undergoing *C. irritans* infection [7]. Surprisingly, *il6* expression was only up-regulated in HK, but not in the infected gills, though it was up-regulated at the site of infection with the enteric parasite *E. leei* [24]. Interestingly, all of the genes related to pro-inflammatory cytokines that were analysed were up-regulated in the HK, suggesting that infection promotes an inflammatory process in which the HK is involved as a source of immune cells but also as a secondary organ, as previously described upon a bacterial infection [43]. Thus, the increase in the HK of *ncf4* gene expression and the decrease in *tryp*, *csf1r,* and several lymphocyte gene markers (*tcrb*, *cd8a* and *ight*) after 2 days of the beginning of the mortalities suggest active granulopoietic activity and/or the recruitment of AGs together with the mobilization of several types of lymphocytes to the peripheral tissues. Gilthead seabream AGs can act as antigen-presenting cells [43]. The decrease in the *mhc2a* transcription in the HK supports the idea of large-scale leukocyte mobilization from the HK and could be also related to the increased granulopoiesis in the HK with many immature and not fully functional AGs. Regarding B lymphocytes, in grouper (*Epinephelus coioides*), both *ighm* and *ight* expression levels decrease in the HK of fish infected with *C. irritans* [49], while in rainbow trout infected with *I. multifiliis,* these genes only started to be up-regulated at 14 days post-infection [50]. These data suggest that the specimens under study in this severe natural outbreak of *C. irritans* only developed an early innate immune response with a strong inflammatory response and granulocyte mobilization but without the local generation of specific B responses, suggesting that this is the first contact with the parasite in their history. Although deeper studies using specific antibodies against different immune cells and cell proliferation analysis are mandatory to corroborate these suggestions, our expression data represent a step forward in the comprehension of the immune response upon the presence of a gill parasite. 

On the other hand, cell-mediated cytotoxicity (CMC) is an important defence for fish and comprises non-specific (depending on non-specific cytotoxic cells, NCCs) and specific (depending on cytotoxic T-lymphocytes, CTLs) arms. Transcription of the marker for NCCs (*nccrp1*) was up-regulated upon infection in the gills. NCCs appear to have an important role in the defence against parasites such as *I. multifiliis* or *Tetrahymena pyriformis* [51]. As in our work, NCCs are suggested to be accumulated in *Cryptocaryon*-infected tissues in grouper *(E. coioides)* and participate in the immune response when this parasite invades its host [52]. In fact, gilthead seabream NCC activity is increased upon *E. leei* infection [23] towards the transcription of *nccrp1*, *gzma*, and *perforin* [24]. In addition, all types of T cell markers, including those of CTLs were up-regulated in the intestine of seabream parasitized with *E. leei*, while the gene expression of CTL markers (*tcrb* and *cd8a*) was down-regulated in both gills and in the HK [24]. All of these data point to the importance of both NCCs and CTLs as a local response against *E. leei*. Nevertheless, our data show an impaired mobilization of CTLs from the HK that did not reach the infected gills. It has been documented that the human parasite *Toxoplasma gondii* is able to manipulate the host immunity by the dysregulation of immune-related gene transcription [53]. As such, we might hypothesize that the arrival of CTLs to the gills is disrupted by some unknown *C. irritans* evasion mechanism, which merits further research. Regarding the expression of NCC and CTL effectors, our data only documented an up-regulation of *fasl* in the gills but not of *gzma* or *gzmb*. Although the up-regulation of the granzymes as well as other cytotoxic effectors is documented in turbot infected with *P. dicentrarchi* [54], seabream infected with *E. leei* [24], or in rainbow trout infected with the myxosporean *Ceratonova shasta* [55], we failed to find this pattern. However, we found large-scale up-regulation of the *fasl* gene in the gills of infected seabream, which was also found in turbot infected with *P. dicentrarchi* [51], supporting the relevance of the granule-independent mechanism against parasites in fish [25]. Our data suggest that *C. irritans* promotes local innate CMC activity mediated by NCCs through a granule-independent pathway using FasL and not through a granule-dependent pathway using granzymes. Further studies are mandatory to clearly establish the role of CMC in the immune response upon *C. irritants* infection.

Based on our data regarding leukocyte mobilization and the up-regulation of innate immunity mediators as the most relevant for fighting against *C. irritans* infection, we next analysed several innate immune activities in the serum. Thus, complement, peroxidase, lysozyme, and bactericidal activities were determined. We did not observe differences in the activities between the infected and control fish, except for the bactericidal activity, which increased in the serum of *C. irritans*-infected specimens. In contrast, gilthead seabream specimens undergoing other parasitic infections such as *E. leei* showed increased complement and peroxidase activity during infection [27]. We analysed the bactericidal activity against *Vibrio harveyi* due to the impossibility to do so against *C. irritans*, and the enhancement that was observed might be due to an increase in different types of AMPs, as they play a major role in bactericidal activity [30]. This is a reason to analyse the gene expression of several AMPs in the gills and HK and the protein level of hepcidin and NK-lysin in the serum. We found that the lysozyme activity and hepcidin and NK-lysin levels in the serum were unmodified upon infection. Taking into account that the AMP activity might be variable against different biological targets, such as *Vibro harveyi* and *C. irritans*, we cannot discard the biological activity of the lysozymes, hepcidin, and NK-lysin against *C. irritans*. In fact, large-scale up-regulation of *lyz* and *hamp* gene expression in the gills occurred upon infection. Similarly, the up-regulation of *hamp* transcription in the gills has also been described in barramundi (*Lates calcarifer*) suffering from *C. irritants* infection [17]. In addition, the expression levels of all of the AMPs analysed in the HK were down-regulated upon infection. All of these data point to a local immune response based on AMP production in the gills. Interestingly, NKL, an AMP that is mainly produced by CTLs and natural killer (NK) cells [56], is stored in cytolytic granules and is released upon stimuli and has an important role in the CMC [57]. In our study, we observed very low levels of *nkl* expression in the gills and HK compared to control fish levels. This fact also points to the idea that CMC activity is not mediated by granule-dependent pathways. Interestingly, an early up-regulation of the *nkl* expression levels in the gills of yellow croaker infected with *C. irritans* was observed followed by a down-regulation after 24 h of infection [21]. All of these data once gain point to a local immune response in the gills based on innate immune effectors and a poor specific immune response, probably due to an impairment in the recruitment of specific immune cells. Taking the fact that we only analyzed selected genes using qPCR into account, we cannot broaden our analysis far from the immune responses in which the genes that were analyzed have a key role. For a complete overview of the effect of *C. irritans* in gilthead seabream physiology and immune response, massive transcriptomic studies are mandatory. However, our data point to the fact that local inflammation, leukocyte recruitment, innate CMC activity mediated by NCCs through a granule-independent pathway, and some AMPs had a key role in the response against a natural outbreak of *C. irritans* in gilthead seabream.

## 4. Materials and Methods

### 4.1. Animals

Gilthead seabream specimens were bred and kept at the facilities of the Oceanographic Centre of Murcia, (IEO-CSIC) in Mazarrón (Spain). Fish from the same batch of hatched eggs were placed in different 7 m^3^ tanks with a natural water temperature and photoperiod and an open flow-through circuit with suitable aeration. The environmental parameters, mortality, and food intake were recorded daily.

The handling of the specimens was always performed in accordance with the Guidelines of the European Union Council (2010/63/UE) and the Bioethical Committees of the IEO-CSIC (reference REGA ES300261040017).

### 4.2. Natural Outbreak and Sampling

In the southeast of the Mediterranean Sea, where our facility is located (37°34′36.0″ N 1°14′02.3″ W), frequent outbreaks of *Cryptocaryon irritans* occur when temperatures increase to over 20 °C. In our case study (28 July 2019; 26 ± 0.3 °C), in a stock of gilthead seabream specimens of 102 ± 19 g of body mean weight (bw), we observed very severe and the sudden mortality of 92.5% of the specimens over the course of one day (day 0), which reached 100% after 8 days. Samples were taken, and fresh gills were observed with a binocular loupe, finding *C. irritans* trophonts as expected. Two days after the massive mortalities, six live fish were sampled. Six other fish from the same batch of hatched eggs (same age) located in other tanks with temperatures controlled at 20 °C, in which neither mortalities nor parasites in fresh gills were observed, served as the control group.

All of the specimens were sacrificed using an overdose of clove oil (40 μL/L), and the fish were weight. Blood samples were collected from the caudal vein with an insulin syringe, and the specimens completely bled, decapitated, and their gills and head kidney (HK) were removed. Serum samples were obtained via the centrifugation of the blood (10,000× *g*, 10 min, 4 °C), and they were immediately frozen and stored at −80 °C. Fragments of the HK and gills were removed and immediately frozen in TRIzol^®^ Reagent (Life Technologies, Carlsbad, CA, USA) at −80 °C until they were used for RNA isolation. A fragment of gills of each specimen was processed for light microscopy analysis.

### 4.3. Light Microscopy and Immunohistochemistry (IHC)

Gill fragments were fixed with Bouin’s solution for 16 h. Then, they were dehydrated using increasing solutions of ethanol in water (70%, 96%, and twice 100% baths) for 60 min each, cleared with two baths in xylene substitute (Sigma-Aldrich, St. Louis, MO, USA; 30 min in each bath), and embedded in paraffin (Paraplast Plus, Sherwood Medical, Sherwood, Athy, Ireland) for 16 h. Then, sections that were 5 μm thick were cut. After dewaxing and rehydration in decreasing solutions of ethanol in water, some sections were stained with haematoxylin–eosin, while others were subjected to an indirect immunohistochemistry method using the monoclonal antibody D2, which specifically binds to seabream acidophilic granulocytes (AGs) and was previously characterized [42]. Briefly, the rehydrated sections were incubated twice in peroxidase quenching solution (H_2_O_2_ in methanol, 1:9) for 20 min at room temperature (RT) to eliminate endogenous peroxidase activity. Afterwards, sections were rinsed in 0.1 M phosphate buffer (PBS) and in PBT (0.01 M PBS; 0.01% bovine serum albumin (BSA; Sigma-Aldrich); 0,05% Tween 20 (Sigma-Aldrich, St. Louis, MO, USA)) for 5 min each. To prevent non-specific antibody binding, the sections were blocked with 5% BSA in PBS for 30 min at RT, and they were then rinsed in PBT for 5 min twice. Afterwards, the sections were incubated with D2 mAb with an optimal dilution of 1:100 for 1 h at RT. After washing the sections in PBT, they were incubated with an anti-mouse IgG (whole molecule) peroxidase conjugated antibody (Sigma-Aldrich) at the optimal dilution of 1:100 for 1 h at RT. The sections were then washed in PBT, and the peroxidase activity developed by incubation with 3,3′-diaminobenzidine tetrahydrochloride (DAB, SigmaFast DAB) according to the manufacturer’s instructions. The slides were examined with an Eclipse E600 light microscope (Nikon, Minato-ku, Japan). The images were obtained with an Olympus SC30 digital camera (Olympus soft imaging solutions).

### 4.4. Hepcidin and NK-Lysin Quantification by ELISA

Rabbit polyclonal antisera against gilthead seabream hepcidin and NK-lysin were produced (GenScript). For this, from putative protein sequences of seabream hepcidin (XP_030248644) and NK-lysin (XP_030299163.1), antigenic determinants in the C-terminus of hepcidin (CPSRVREKRQSHISM), and NK-lysin (CRSDVDAKFEMIDYP) were predicted, and the synthetic peptides were chemically synthetized and used to immunize the rabbits (GenScript Biotech, Leiden, The Netherlands).

Quantification of serum hepcidin and NK-lysin AMPs was performed using an indirect enzyme-linked immunosorbent assay (ELISA) with the obtained specific antisera. Progressive serial dilutions of gilthead seabream serum (1:50, 1:100, 1:500, and 1:1000) and rabbit anti-hepcidin and anti-NK-lysin sera (1:500, 1:1000, 1:2000, 1:5000) were used to determine the linearity of the serum dilution curve and the specificity of the antibodies. In addition, a pre-absorbed control was performed using the synthetic peptide to determine the specificity of the reaction. For that, each primary diluted antiserum (anti-NK-lysin: 1/1000; anti-hepcidin: 1/2000) was incubated with its respective synthetic peptide at 20 nmol for 16 h at 4 °C before using it in the ELISA assay. These data are included in Supplementary Figure S1.

Specific detection of NK-lysin and Hepcidin was then performed in the serum from the control and *C. irritans*-parasitized samples. For this, seabream serum samples were diluted (1:1000) in coating buffer (100 mM Bicarbonate/Carbonate pH 9.6), disposed in flat bottomed 96-wells (Thermo-Fisher Scientific, Waltham, MA, USA), and incubated overnight at 4 °C. Afterwards, the samples were washed four times in PBT. The non-specific unions were blocked with PBS containing 3% BSA for 2 h at RT. After washing with PBT, the primary antiserum at the optimal dilution (anti-NK-lysin: 1/1000; anti-hepcidin: 1/2000) were incubated for 1 h at RT and were then with an anti-rabbit IgG conjugated with peroxidase 1/1000 for 1 h at RT. The reaction was developed by adding 10 M 3,3′,5,5′-tetramethylbenzidine (TMB; Sigma-Aldrich) containing 0.0015% hydrogen peroxide (Scharlau, Barcelona, Spain) over 10 min and was stopped with 2 M sulphuric acid. Absorbance was measured at 450 nm using a plate reader (Multiskan GO; Thermo Scientific, Waltham, MA, USA). Negative controls with no sample or primary antiserum were always included. NK-lysin and hepcidin synthetic peptides were used as positive controls.

### 4.5. Analysis of Gene Expression by Real-Time PCR

Total RNA was isolated from TRIzol Reagent frozen independent samples (*n* = 6/group) following the manufacturer’s instructions and was treated with amplification grade DNase I (1 unit/µg RNA; Invitrogen, Waltham, MA, USA). The SuperScript IV Reverse Transcriptase (Invitrogen) was used to synthesize the first strand cDNA with the oligo-dT18 primer from 1 µg of total RNA at 50 °C for 60 min. Real-time PCR was performed with a Quant Studio 5 instrument (Applied Biosystems) using SYBR Green PCR Core Reagents (Applied Biosystems) to determine the gene expression coding for relevant-immune molecules in every individual sample: (i) pro-inflammatory molecules such as interleukin-1β (*il1b*), interleukin-6 (*il6*), and the CXC chemokine *il8*; (ii) anti-microbial peptides NK-lysin (*nkl*), β-defensin (*bdef*), lysozyme (*lyz*), and hepcidin (*hamp*); (iii) innate immune cell markers including NADPH oxidase subunit p40phox (*ncf4*), colony-stimulation factor-1 receptor (*csf1r*), myeloperoxidase (*mpo*), tryptase (*tryp*), and the major histocompatibility complex II alpha (*mhc2a*); (iv) lymphocyte receptors such as the T cell receptor (*tcrb*), CD4 receptor (*cd4*), CD8 receptor alpha-chain (*cd8a*), the heavy chain of immunoglobulins M (*ighm*), and T (*ight*); or (v) cell-mediated cytotoxicity molecules such as non-specific cytotoxic cell receptor 1-type (*nccrp1*), fas ligand (*fasl*), granzyme A (*gzma*), and granzyme B (*gzmb*). The reaction mixtures were incubated at 95 °C for 10 min followed by 40 cycles of 15 s at 95 °C, 1 min at 60 °C, and finally 15 s at 95 °C, 1 min at 60 °C, and 15 s at 95 °C. For each mRNA, gene expression was corrected by the *elongation factor 1 alpha* (*ef1a*) expression in each sample and was expressed as 2^−ΔCt^, where ΔCt is determined by subtracting the *ef1a* Ct value from the target Ct [58]. The primers used are shown in Supplementary Table S1. Negative controls with no template were always included in the reactions.

### 4.6. Antimicrobial Activities in Serum

Several antimicrobial activities were determined in the gilthead seabream serum as described below. The total protein in the serum was measured using the Bradford method [59].

#### 4.6.1. Natural Haemolytic Complement Activity

The activity of the alternative complement serum pathway was assayed using a suspension of red blood cells (SRBC) from fresh pig blood as targets according to a protocol that was previously described [60]. Equal volumes of SRBC suspension (6%) in phenol red-free Hank’s balanced salt solution (HBSS, Gibco) containing Mg^2+^ and ethylene glycol tetra acetic acid (EGTA, Sigma-Aldrich) were mixed with serially diluted seabream serum to provide final serum concentrations ranging from 10% to 0.078%. After incubation for 90 min at RT, the samples were centrifuged at 400× *g* for 10 min at 22 °C to avoid unlysed erythrocytes. The relative haemoglobin content of the supernatants was assessed by measuring their optical density (OD) at 550 nm on a plate. A blank was prepared by replacing the sample with HBSS to measure spontaneous haemolysis. A positive control was prepared by replacing the sample with distilled water in order to know the maximum haemolysis value.

The degree of haemolysis (Y) was estimated, and the lysis curve for each specimen was obtained by plotting Y/(1−Y) against the volume of the serum added (mL) on a log–log scaled graph. The volume of serum producing 50% haemolysis (ACH_50_) was determined, and the results were represented as ACH_50_ units/mL of serum.

#### 4.6.2. Lysozyme Activity

The lysozyme activity in the serum was measured using a modified turbidimetric method that was previously described [61]. Firstly, 100 μL of seabream serum diluted to a ratio of 1:2 with 0.01 M PBS at pH 6.2 were placed in flat-bottomed 96-well plates (Nunc) in duplicate. Afterwards, 100 μL of 0.3 mg/mL of freeze-dried *Micrococcus lysodeikicus* (Sigma-Aldrich) in phosphate-citrate buffer (0.13 M Na_2_HPO_4_; 0.11 M citric acid; 0.015 M NaCl, pH 6.2) was added as a lysozyme substrate. Changes in absorbance at 450 nm were measured immediately every 30 s for 30 min at 25 °C in a plate reader. One unit of lysozyme activity was defined as a reduction in the absorbance of 0.001/min. The lysozyme units that were present in the serum were obtained from a standard curve ranging from 20 to 0 units/mL made with hen egg white lysozyme (HEWL, Sigma-Aldrich) and the results were expressed as units/mL of serum.

#### 4.6.3. Bactericidal Activity

The pathogenic marine bacteria *Vibrio harveyi* (Vh) (strain Lg 16/100) was grown in agar plates at 25 °C in tryptic soy agar (TSA, Sigma-Aldrich). Then, fresh single colonies of 1–2 mm were diluted in 5 mL of tryptic soy broth (TSB; Sigma-Aldrich), cultured for 16 h at 25 °C on an orbital incubator at 200–250 rpm, and adjusted to 10^8^ bacteria/mL of TSB. The absorbance of the bacteria cell cultures was measured at 600 nm and was used to determine the concentration based on growth curves.

The antibacterial activity of the serum was determined by evaluating the bacterial growth curves of Vh using a method that was previously described [62]. Aliquots of 10 μL of seabream serum samples were placed in a flat-bottomed 96-wells plate, mixed with 10 μL of the bacterial dilution (1/10), and incubated 120 min at RT. Afterwards, 150 μL of TSB were added to each well, and the OD measured at 620 nm every 30 min over the course of 38 h at 25 °C. A negative control (0% bactericidal activity, 0% growth) was prepared by replacing the sample and bacteria solution with TSB, while a positive control (0% bactericidal activity, 100% growth) was prepared by replacing the sample with TSB. Bactericidal activity was expressed as the % of bacterial growth inhibition.

#### 4.6.4. Peroxidase Activity

The peroxidase activity in serum was measured according to a protocol previously described [63]. Firstly, 5 μL of seabream serum diluted with 45 μL of Hanks buffer without Ca^2+^ nor Mg^2+^ at pH 7.2 was placed in flat-bottomed 96-well plates in triplicate. As a substrate, 100 μL of 10 mM TMB solution with 0.015% of H_2_O_2_ was added. This chromogenic reaction was stopped after 10 min incubation with 50 μL of 2 M H_2_SO_4_. Then, OD was measured at 450 nm using a plate reader. Wells with buffer but not with sample were used as blanks. One unit was defined as the amount of activity producing an absorbance change of 1, and the activity was expressed as U/mL of serum.

### 4.7. Statistical Analysis

All data are presented as mean ± standard error of the mean (SEM). Statistical differences between groups were analysed accordingly with Student’s *t* test. The normality of the distributions and the homoscedasticity of the variances were tested through the Shapiro–Wilk and Levene tests, respectively. Non-normally distributed data were log-transformed prior to analysis. When the data did not meet parametric assumptions, a U-Mann–Whitney test was used. Statistical analyses were conducted using SPSS 24 software. The minimum level of significance was fixed at 0.05 (*p* ≤ 0.05).

## 5. Conclusions

In conclusion, a severe natural outbreak of *C. irritans* in gilthead seabream produced complete mortality and alteration of the gills structure. Our data point to active leukocyte mobilization in HK and gills, where inflammation, granulocyte infiltration, and probably the innate CMC and AMP responses seem to be crucial for the defense of gilthead seabream upon *C. irritans* infection.

## Data Availability

Data are contained within the manuscript.

## Supplementary Materials

The following are available online at https://www.mdpi.com/article/10.3390/ijms23020937/s1.
