# Supplementary material for: Severe Natural Outbreak of Cryptocaryon irritans in Gilthead Seabream Produces Leukocyte Mobilization and Innate Immunity at the Gill Tissue
Source: Int J Mol Sci. 2022 Jan 15;23(2):937. doi: 10.3390/ijms23020937 (PMC8780452; doi:10.3390/ijms23020937)
Supplement: Supplementary file 1 [file ijms-23-00937-s001.zip › Supplementary table - copia.pdf]

Supplementary Table S1. Information about the studied genes and primer sequences used in this study.

Protein name	Gene name	Accession number	Sequence (5'→3')
Elongation factor 1 alpha	*ef1a*	AF184170	F: CTTCAACGCTCAGGTCATCAT<br>R: GCACAGCGAAACGACCAAGGGGA
Myeloperoxidase	*mpo*	FM148574	F: TTGGTCCAGACATCCTCG<br>R: ATGGGCAAAGCGGTAG
Tryptase-like	*tryp*	NC_044209	F: AAATGTGAAACTGCCCAGCG<br>R: GATCCGGCAGTGGAGCATTA
Colony stimulation-factor 1 receptor	*csf1r*	AM050293	F: ACGTCTGGTCCTATGGCATC<br>R: AGTCTGGTTGGGACATCTGG
NADPH oxidase subunit p40phox	*ncf4*	AM749961	F: GCGGAGTTGAACCTGAAGAG<br>R: TCACCTTCTGTGTCGCTGTC
Major histocompatibility complex-II alpha	*mhc2a*	DQ019401	F: CTGGACCAAGAACGGAAAGA<br>R: CATCCCAGATCCTGGTCAGT
T-cell receptor beta chain	*tcrb*	AM261210	F: AAGTGCATTGCCAGCTTCTT<br>R: TTGGCGGTCTGACTTCTCTT
T-cell antigen cluster of differentiation 4	*cd4*	AM849812	F: TGTCACAGTCATCGTGCTCC<br>R: GCCTGGGGTGTCTCATCTTC
T-cell antigen cluster of differentiation 8a	*cd8a*	AJ846849	F: CTGTCCTCCGCTCATACTGG<br>R: TTGTAATGATGGGGGCATCT
Heavy chain of immunoglobulin M	*ighm*	JQ811851	F: CAACATGCCCAATTGATGAG<br>R: GGCACGACACTCTAGCTTCC
Heavy chain of immunoglobulin T	*ight*	FM145138	F: TGGCAAATTGATGGACAAAA<br>R: CCATCTCCCTTGTGGACAGT
Interleukin-1 beta	*il1b*	AJ277166	F: GGGCTGAACAACAGCACTCTC<br>R: TTAACACTCTCCACCCTCCA
Interleukin-6	*il6*	AM749958	F: AGGCAGGAGTTTGAAGCTGA<br>R: ATGCTGAAGTTGGTGGAAGG
Interleukin-8	*il8*	AM765841	F: GCCACTCTGAAGAGGACAGG

			R: TTTGGTTGTCTTTGGTCGAA
NK-lysin	*nkl*	MN240490	F: CGCACCTCGGAGAACTGATT
			R: TCCACGTCGCTTCGGTAAAA
β-defensin	*bdef*	FM158209	F: CCCCAGTCTGAGTGGAGTGT
			R: AATGAGACACGCAGCACAAG
Lysozyme	*lyz*	AM749959	F: CCAGGGCTGGAAATCAACTA
			R: CCAACATCAACACCTGCAAC
Hepcidin	*hamp*	CB184616	F: GCCATCGTGCTCACCTTTAT
			R: CTGTTGCCATACCCCATCTT
Non-specific cytotoxic cell receptor-type 1	*nccrp1*	AY651258	F: ACTTCCTGCACCGACTCAAG<br>R: TAGGAGCTGGTTTTGGTTGG
Fas ligand	*fasl*	ENSSAUT000100	F: GCCACTTTGCCCGAACAATT
			R: GGTGGGGCAGTAAATCACCA
Granzyme A	*gzma*	MK568066	F: TCCCTGCTATGATGCAACTG
			R: ATTTCACCGTCTTGGTTTGC
Granzyme B	*gzmb*	AM957224	F: GAAACAAAGGAACGGGTCAA
			R: GAGCTGTCCATCTTTTGCTTG
