# Supplementary figures and images for: Severe Natural Outbreak of Cryptocaryon irritans in Gilthead Seabream Produces Leukocyte Mobilization and Innate Immunity at the Gill Tissue
Source: Int J Mol Sci. 2022 Jan 15;23(2):937. doi: 10.3390/ijms23020937 (PMC8780452; doi:10.3390/ijms23020937)

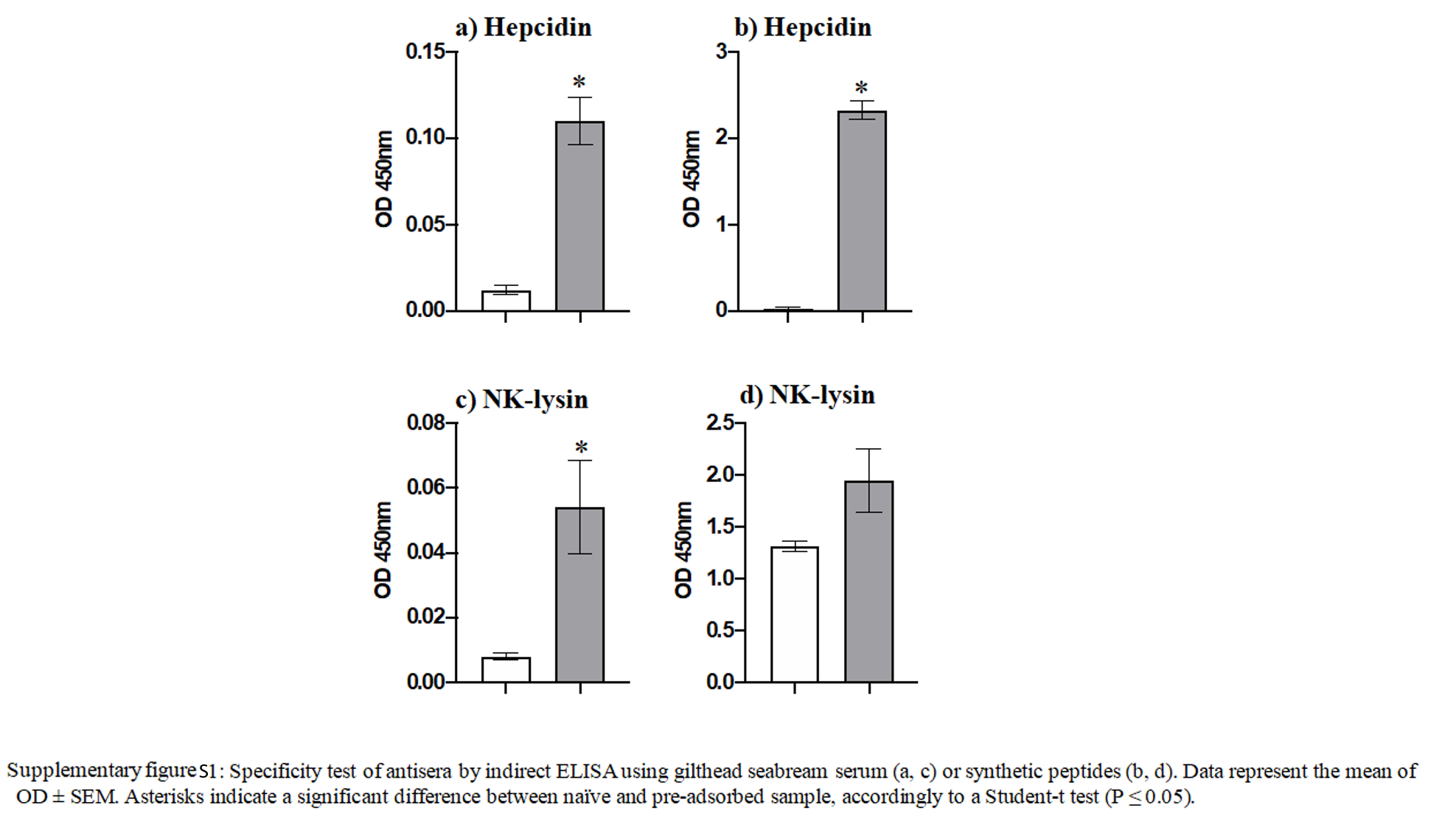

Supplement: Supplementary file 1 [file ijms-23-00937-s001.zip › Supplementary figure S1 - copia.png]
